# Utility of the 4-meter backward walking speed test in older adults with neurodegenerative diseases

**DOI:** 10.1007/s11845-026-04424-w

**Published:** 2026-05-08

**Authors:** Kubra Altunkalem Seydi, Derya Kaya, Idil Yavuz, Alev Cam Mahser, Feyza Mutlay, Fatma Sena Dost, Ahmet Turan Isik

**Affiliations:** 1 Department of Geriatric Medicine, Ministry of Health Edirne Sultan Murat 1 State Hospital, Edirne, Turkey; 2 Geriatric Sciences Association, Izmir, Turkey; 3 Unit for Aging Brain and Dementia, Department of Geriatric Medicine, Faculty of Medicine, Dokuz Eylul University, Izmir, Turkey; 4 Department of Statistics, Faculty of Science, Dokuz Eylul University, Izmir, Turkey; 5 Department of Internal Medicine, Division of Geriatrics, Ministry of Health Eskisehir City Hospital, Eskisehir, Turkey; 6 Department of Geriatric Medicine, Faculty of Medicine, Çanakkale Onsekiz Mart University, Çanakkale, Turkey

**Keywords:** Backward walking, Gait, Alzheimer’s disease, Parkinson’s disease

## Abstract

**Background and aim:**

Less is known about backward walking speed (BWS) in older adults. This study aims to establish a cutoff value for BWS to distinguish cognitively impaired individuals from healthy controls and to assess the association between backward walking slowing and risk of neurodegenerative diseases.

**Methods:**

389 older patients, grouped into cognitively healthy (CH), amnestic mild cognitive impairment (aMCI), Alzheimer’s disease (AD), Parkinson’s disease (PD), and non-AD groups. BWS was measured using a standardized 4-meter protocol. Cognitive and functional status were evaluated via the comprehensive geriatric assessment. ROC analysis was used to determine the diagnostic threshold for BWS, and multinomial logistic regression was used to assess the associations.

**Results:**

A BWS cutoff of 0.395 m/s effectively distinguished CH individuals from those with neurodegenerative diseases (AUC=0.723). After adjusting for age, each 0.1 m/s decrease in BWS was associated with 22% increased odds of having aMCI, 37% increased odds of AD, 28% increased odds of PD, and 59% increased odds of having non-AD dementia. BWS showed a positive association with global cognitive scores in patients with CH and aMCI (p< 0.05) and with semantic verbal fluency scores in those with AD and aMCI (*p*< 0.05).

**Conclusion:**

BWS is associated with an increased risk of neurodegenerative diseases, which can cause cognitive impairment, and may help indicate individuals at risk of cognitive decline.

## Introduction

Walking speed (WS) may serve as an indicator of well-being, cognitive and physical functionality, and mortality risk in older adults [1, 2]. It is referred to as the sixth vital sign [3]. Walking is a complex biomechanical activity that requires the integration of a synchronized neuromusculoskeletal system, along with the coordination, interaction, and adaptation of various cognitive processes, including attention, planning, memory, and executive functions [4]. WS can decline with advancing age, and the decline associated with aging is more noticeable during backward walking (BW) than during forward walking (FW) [5]

Backward walking is a complex motor task that requires greater cognitive resources, particularly proprioception and attention, due to reduced visual feedback [6]. BW also challenges postural stability, tests an individual’s confidence in maintaining balance, and requires spinal and pelvic stabilization to sustain dynamic equilibrium. As such, BW is recognized as a postural balance stimulator and a potential facilitator of neuroplasticity enhancements [7, 8]. Deficits in BW may be more pronounced than those observed in FW, particularly in the context of neurodegenerative diseases. It appears to be more sensitive to the effects of neurodegenerative conditions and may be impacted earlier in the disease trajectory, as evidenced in individuals with mild to moderate Parkinson’s disease (PD) [9]. Considering these factors, the impairment in BW speed (BWS) may reflect early involvement of the frontal-subcortical and parietal networks, which play a role in neurodegenerative diseases [10]. However, research lacks emphasis on what happens to the effect of BWS when it comes to the risk of mild cognitive impairment (MCI), Alzheimer’s disease (AD), PD, and/or non-AD dementias. To the best of our knowledge, no investigation has explored the effect of BWS on the risk of the aforementioned neurodegenerative diseases, nor has it examined the cutoff value for BWS in older adults, both with and without cognitive impairment (CI).

Currently, research is evolving to identify these neurodegenerative diseases as early as possible to benefit from monoclonal treatments [11], potential immunomodulatory drugs [12], and/or multidomain prevention programs [13]. In parallel to these efforts, tools that are safe, cost-effective, and non-invasive are crucial to find the individuals in their prodromal, preclinical phases or early clinical stages. BWS is easier to apply and interpret than other gait parameters, such as stride length variability and stride time variability, and has a potential relationship with neurodegeneration [14, 15]. Therefore, a defined threshold for BWS enables clinicians to distinguish individuals who may require further cognitive or functional evaluation, facilitates standardized interpretation across settings, and supports early screening of those at increased risk for neurodegenerative diseases. In this respect, examining BWS and risk of diseases such as MCI, AD, PD and non-AD dementias can be clinically meaningful to help improve diagnosis and outcome prediction, and provide information to guide assessment of treatments’ efficacy in those neurodegenerative diseases.

Hence, the aim of this research is twofold: (i) to establish a cutoff value for BWS to distinguish cognitively impaired individuals from healthy ones and (ii) to assess the association between BW slowing and risk of various neurodegenerative diseases.

## Materials and methods

### Study design

In this cross-sectional study, we evaluated 389 patients who attended our geriatric outpatient clinics between September 2022 and October 2023. The study included patients aged 65 and older who met the following criteria: the ability to stand up from a seated position independently or with the aid of a chair arm no more than once, the ability to understand two-step commands, and a Mini-Mental State Examination (MMSE) score of ≥ 10. This study was conducted in accordance with the Declaration of Helsinki and approved by the local ethics committee (Ethics Committee number: 2019/07–57, date: 27.03.2019). The written consents were provided by patients or their guardians.

### Participant diagnostics

MCI due to AD was diagnosed according to the criteria established by the National Institute on Aging and the Alzheimer’s Association (NIA-AA) Workgroup. The core clinical features include: (a) a cognitive concern reflecting a change in cognition, reported by the patient, an informant, or observed by a clinician; (b) CI in one or more cognitive domains preferably relative to appropriate normative data for that individual; (c) preservation of independence in functional abilities; (d) not demented [16, 17].

The diagnosis of AD was made in accordance with the NIA-AA criteria, which require documented evidence of a gradual decline in memory and other cognitive domains that interfere with independence in daily activities, supported by clinical history, neuropsychological assessment, and the exclusion of alternative medical or psychiatric causes [18].

Idiopathic PD was diagnosed according to the Movement Disorder Society Clinical Diagnostic Criteria for Parkinson’s Disease. These criteria define parkinsonism as the presence of bradykinesia, in combination with either rest tremor or rigidity. The diagnosis of PD requires the absence of absolute exclusion criteria, the presence of at least two supportive criteria, and the absence of red flags that would suggest an alternative diagnosis [19].

Non-AD dementia was diagnosed according to the Diagnostic and Statistical Manual of Mental Disorders, Fifth Edition (DSM-5) criteria for Major Neurocognitive Disorder, which require evidence of significant cognitive decline from a previous level of performance in one or more cognitive domains, interference with independence in everyday activities, and that another mental disorder does not better explain these deficits [20]. The non-AD dementia group included heterogeneous etiologies such as dementia with Lewy bodies, Parkinson’s disease dementia, frontotemporal dementia and atypical parkinsonian syndromes.

### Exclusion criteria

Patients were excluded from the study if they met any of the following criteria: low visual acuity impairing mobility, history of stroke, severe osteoarthritis or neuromuscular disorders significantly affecting movement, complete dependence in activities of daily living, severe hearing loss, superficial or deep sensory deficits on examination, or acute medical conditions. Patients who had undergone orthopedic surgery within the past year, those with a history of alcohol or substance abuse, and individuals diagnosed with delirium or psychotic disorders were also excluded.

### Comprehensive geriatric assessment

Global cognition was evaluated with the Montreal Cognitive Assessment (MoCA) (patients with 9 years or more of education received) [21] and the MMSE [22]. MoCA scores were converted to MMSE scores as needed [23]. This conversion was applied to 159 participants. The Yesavage Geriatric Depression Scale (YGDS) [24] was used for depression screening. Verbal fluency (VF) tests used in this study included phonemic and semantic VF [25]. The participants were asked to name as many animals as possible for semantic VF and to name as many words as possible that begin with the letter “K” for phonemic VF within 60 s. The participants were instructed to avoid using proper nouns and words that share the same beginning sound but have different endings; errors of this nature were excluded from the total count of correct words. The Lawton-Brody Instrumental Activities of Daily Living (IADL) [26] and Barthel Index for Activities of Daily Living (BADL) [27] were used to evaluate functionality. The Tinetti Performance-Oriented Mobility Assessment (POMA) [28], Timed Up and Go Test (TUGT) [29], and 4-meter walk speed test [30] were used to assess balance and gait functions. Sociodemographic features and comorbidities were also collected [31].

### Backward walking speed test assessment

The BWS test was administered using a standardized 4-meter walk protocol adapted from the British Columbia Frailty Guidelines [32]. Participants were instructed to walk backward at their usual pace, simulating natural daily movement. The test was conducted in a well-lit hallway on a flat, dry surface. A total distance of 6 m was demarcated, comprising a 1-meter acceleration zone, a central 4-meter testing zone, and a 1-meter deceleration zone to ensure consistent walking speed within the measurement segment. Timing began with the first footfall after the 0-meter line and stopped with the first footfall after the 4-meter mark. The participants wore their usual footwear and were allowed to practice the task once before the actual trials. The BWS test was repeated twice, and the mean of the obtained measurements was used in the analyses. Gait speed was calculated in meters per second (m/s) via the following formula: speed = distance/time. No verbal encouragement was provided during the trials to minimize performance bias. For safety, a trained examiner walked alongside the participant without providing physical assistance [32].

### Statistical analysis

For quantitative variables, means and standard deviations $\left(\overline x\pm s\right)$ are reported, and comparisons across diagnostic groups were conducted using ANOVA when assumptions were met; otherwise, the Kruskal-Wallis test was employed. For qualitative variables, frequencies and percentages (n, (%)) are presented, with group comparisons performed using Chi-squared tests. Assuming an average of 1,500 first-time patient admissions annually to the geriatric outpatient clinic and considering a prevalence rate of 50% (a standard estimate for conditions with unknown frequency), the required sample size was calculated to be a minimum of 306 patients. This calculation was based on a 5% margin of error and a 95% confidence level. To determine threshold values of BWS for distinguishing cognitively impaired individuals from healthy controls, Receiver Operating Characteristic (ROC) curves were generated. The area under the curve (AUC), along with corresponding sensitivity and specificity values, was used to evaluate diagnostic performance. Correlations between BWS and cognitive as well as functional assessment scores across diagnostic subgroups were examined using Spearman’s rho. The relationship between BWS and diagnostic group classification was further assessed using simple multinomial logistic regression models, with the CH subgroup designated as the reference category. Regression coefficients, odds ratios (ORs), and confidence intervals were reported for each 0.1-unit change in walking speed. As age differed significantly across diagnostic groups, multiple multinomial logistic regression analyses adjusted for age were also performed and interpreted accordingly. A significance level of 0.05 was used for all statistical analyses.

## Results

### Demographic and clinical characteristics

The study included a total of 389 participants, categorized into five diagnostic subgroups: CH; *n* = 239, aMCI; *n* = 32, AD; *n* = 47, PD; *n* = 30, and non-AD; *n* = 41. The mean age differed significantly across the groups (*p* < 0.001). Significant differences were observed among the diagnostic groups for MMSE, BADL, IADL, POMA scores, FWS, and BWS. However, scores on the YGDS did not differ significantly across groups (*p* = 0.214). Table 1 presents the comparative sociodemographic and clinical characteristics of the diagnostic groups.

**Table 1:** Demographic, clinical, and comprehensive geriatric assessment features of the participants

	CH (*n* = 239)	aMCI (*n* = 32)	AD (*n* = 47)	PD (*n* = 30)	Non-AD (*n* = 41)	*P* value
Demographics						
Age*	71.21 ±7.09	75.28 ±7.04	75.70 ±5.81	73.13 ±9.04	74.39 ±5.91	**< 0.001**
Gender *(Female)(%)*	158 (66.1)	20 (62.5)	35 (74.5)	14 (46.7)	25 (61)	0.149
Comorbidities (%)						
HT	155 (65.7)	22 (68.8)	31 (66)	21 (70)	22 (53.7)	0.572
CAD	29 (12.2)	5 (15.6)	7 (14.9)	5 (16.7)	9 (22)	0.559
DM	70 (29.5)	10 (31.3)	19 (40.4)	8 (26.7)	12 (29.3)	0.643
Comprehensive Geriatric Assessment*						
MMSE	27.33 ±1.85	23.63 ±2.11	18.02 ±4.45	26.27 ±2.85	20.24 ±5.80	**< 0.001**
YGDS	4.25 ±3.64	4.03 ±3.02	4.16 ±3.35	3.93 ±4.13	5.92 ±4.53	0.214
BADL	96.55 ±5.61	93.23 ±7.25	88.67 ±13.16	92.10 ±9.93	84.25 ±18.76	**< 0.001**
IADL	21.59 ±2.34	20.10 ±3.42	13.47 ±6.20	19.13 ±3.56	15.85 ±6.40	**< 0.001**
POMA	26.94 ±2.55	26.32 ±2.20	24.98 ±4.87	24.27 ±4.14	21.65 ±6.03	**< 0.001**
TUGT *(sec)*	10.67 ±4.10	11.81 ±4.52	12.89 ±4.92	12.88 ±4.52	16.08 ±7.60	**< 0.001**
FWS *(m/s)*	0.94 ±0.29	0.75 ±0.25	0.75 ±0.26	0.81 ±0.27	0.68 ±0.23	**< 0.001**
BWS *(m/s)*	0.56 ±0.26	0.41 ±0.21	0.36 ±0.19	0.41 ±0.25	0.33 ±0.23	**< 0.001**

*mean ± SD

*AD*: Alzheimer’s Disease; *aMCI*: Amnestic Mild Cognitive Impairment; *BADL*: Barthel Activity Daily Living; *BWS*: Backward walking speed; *CAD*: Coronary Arter Disease; *CH*: Cognitively Healthy; *DM*: Diabetes Mellitus; *FWS*: Forward walking speed; *HT*: Hypertension; *IADL*: Instrumental Activity Daily Living; *MMSE*: Mini-Mental State Examination; *Non-AD*: Non-Alzheimer’s Disease; *PD*: Parkinson’s Disease; *POMA*: Performance-Oriented Mobility Assessment; *TUGT*: Timed Up and Go Test; *YGDS*: Yesavage Geriatric Depression Scale

Bold values denote statistical significance at the *p* < 0.05 level.

### ROC analysis and AUC for the BWS

**Fig. 1:**
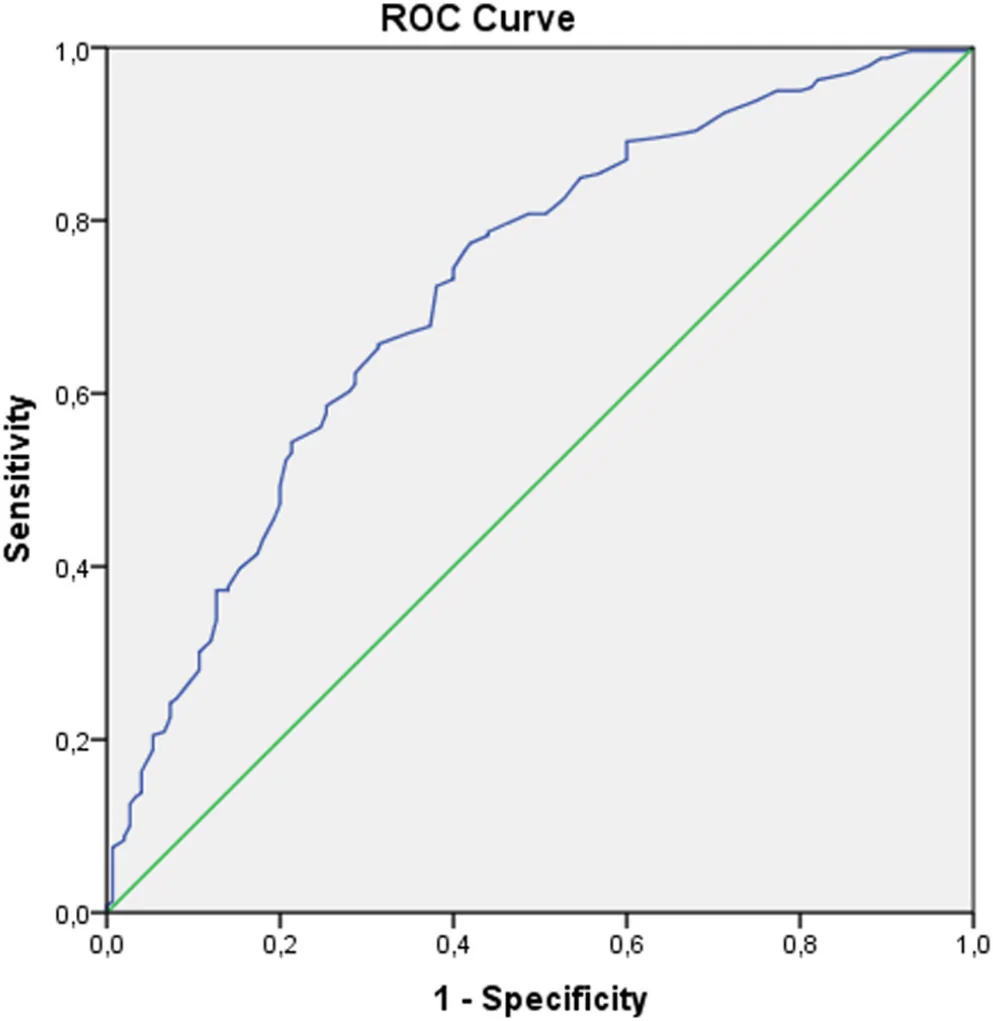
ROC test analysis for identifying a cutoff value for backward walking speed among cognitively healthy participants and the remaining participants. *ROC analysis revealed a sensitivity of 72% and a specificity of 62%, with a cutoff of 0.395 m/s. The area under the ROC curve was 0.732 (CI 95%: *p* < 0.001)

ROC analysis was used to establish a BWS threshold capable of distinguishing individuals with normal cognition from those with neurodegenerative diseases. The analysis identified a BWS cutoff value of 0.395 m/s, yielding an AUC of 0.723, indicative of a moderate discriminatory capacity. This threshold demonstrated a sensitivity of 0.72 and a specificity of 0.62 (Fig. 1). Gender-stratified analysis revealed differences in diagnostic performance. For females, a BWS cutoff value of 0.365 m/s produced an AUC of 0.766, with a sensitivity of 0.70 and specificity of 0.72. For males, a cutoff value of 0.475 m/s resulted in an AUC of 0.684, with sensitivity and specificity values of 0.68 and 0.61, respectively.

### Effect of BWS on participant subgroups

A multinomial logistic regression model was constructed with the participant subgroup as the dependent variable, using the CH group as the reference category and BWS as the predictor. Tables 2 and 3 display the ORs and corresponding regression coefficients for membership in each of the four clinical groups relative to the CH group, based on a 0.1-unit increase in BWS. As lower BWS values are associated with a higher likelihood of CI, the odds ratios were inverted to facilitate clearer interpretation.

**Table 2:** Multinomial logistic regression analysis results for diagnostic groups using backward walking speed

Diagnostic Group	Beta	OR	95% CI, OR	*p*-value
aMCI	-0.262	0.76	0.64–0.91	**0.003**
AD	-0.380	0.68	0.58–0.80	**< 0.001**
Non-AD	-0.474	0.62	0.51–0.75	**< 0.001**
PD	-0.255	0.77	0.65–0.92	**0.004**

*AD*: Alzheimer’s Disease, *aMCI*: Mild Cognitive Impairment, *Non-AD*: Non-Alzheimer’s Disease, *PD*: Parkinson’s Disease

Beta = regression coefficient; OR = odds ratio; 95% CI, OR = 95% confidence interval for the odds ratio;

Bold values denote statistical significance at the *p* < 0.05 level.

**Table 3:** Age-adjusted multinomial logistic regression analysis results for diagnostic groups using backward walking speed

Diagnostic Group	Beta	OR	95% CI, OR	*p*-value
aMCI	-0.194	0.82	0.68–0.98	**0.036**
AD	-0.314	0.73	0.61–0.86	**< 0.001**
Non-AD	-0.462	0.63	0.51–0.77	**< 0.001**
PD	-0.250	0.77	0.64–0.94	**0.010**

*AD*: Alzheimer’s Disease, *aMCI*: Amnestic Mild Cognitive Impairment, *Non-AD*: Non-Alzheimer’s Disease, *PD*: Parkinson’s Disease

Beta = regression coefficient; OR = odds ratio; 95% CI, OR = 95% confidence interval for the odds ratio;

Bold values denote statistical significance at the *p* < 0.05 level.

The model showed a significant effect, where a 0.1-unit decrease in BWS was associated with an increase in the odds of having aMCI by 30%, AD by 46%, PD by 29%, and non-AD dementia by 61% compared with CH individuals (Table 2).

In the adjusted model, which controlled for age differences across groups, a 0.1-unit decrease in BWS increased the odds of having aMCI by 22%, AD by 37%, PD by 28%, and non-AD dementia by 59% compared with CH individuals (Table 3).

### Correlation of BWS with the comprehensive geriatric assessment

The relationships among BWS and cognitive/functional measurements were analyzed across participant subgroups (Table 4). In the CH group, BWS was significantly positively correlated with the MMSE, BADL, IADL, and POMA scores but significantly negatively correlated with the TUGT score. Among patients with aMCI, BWS was significantly positively correlated with the MMSE, semantic VF, BADL, and POMA scores. In the AD group, BWS was significantly positively correlated with the VF test results, as were the BADL, IADL, and POMA scores, but was also significantly negatively correlated with the TUGT results. In both the PD and non-AD groups, BWS had significant positive correlations with BADL, IADL (except for non-AD), and POMA scores, along with a significant negative correlation with TUGT scores. The detailed correlation coefficients are presented in Table 4.

**Table 4:** Correlation analysis of the BWS with comprehensive geriatric assessment outcomes across all diagnostic groups

Variables	CH (*n* = 239)	aMCI (*n* = 32)	AD (*n* = 47)	Non-AD (*n* = 41)	PD (*n* = 30)
MMSE	0.189* (***p* = 0.003)**	0.366* (***p* = 0.039)**	0.229 (*p* = 0.121)	0.031 (*p* = 0.849)	0.223 (*p* = 0.237)
Phonemic VF	0.079 (*p* = 0.427)	0.243 (*p* = 0.446)	0.579** (***p* = 0.038)**	0.007 (*p* = 0.983)	0.290 (*p* = 0.416)
Semantic VF	0.182 (*p* = 0.079)	0.819*** (***p* = 0.002)**	0.612*** (***p* = 0.026)**	0.352 (*p* = 0.262)	0.342 (*p* = 0.573)
BADL	0.301* (***p* < 0.001)**	0.365* (***p* = 0.044)**	0.519** (***p* < 0.001)**	0.522** (***p* = 0.001)**	0.409** (***p* = 0.025)**
IADL	0.191* (***p* = 0.003)**	0.256 (*p* = 0.164)	0.429** (***p* = 0.003)**	0.176 (*p* = 0.276)	0.442** (***p*** ** = 0.015)**
POMA	0.322* (***p* < 0.001)**	0.420** (***p* = 0.019)**	0.473** (***p* = 0.001)**	0.596** (***p* < 0.001)**	0.410** (***p* = 0.024)**
TUGT	-0.546** (***p* < 0.001)**	-0.345 (*p* = 0.062)	-0.667*** (***p* < 0.001)**	-0.692*** (***p* < 0.001)**	-0.650*** (***p* < 0.001)**

*AD*: Alzheimer’s Disease, *aMCI*: Amnestic Mild Cognitive Impairment, *BADL*: Barthel Activity Daily Living, *BWS*: Backward walking speed, *CH*: Cognitively Healthy, *IADL*: Instrumental Activity Daily Living, *MMSE*: Mini-Mental State Examination, *Non-AD*: Non-Alzheimer’s Disease, *PD*: Parkinson’s Disease, *POMA*: Performance Oriented Mobility Assessment, *TUGT*: Timed Up and Go Test, *VF*: Verbal Fluency

Correlation: * weak (|r| = 0.20–0.39), ** moderate (|r| = 0.40–0.59), *** strong (|r| ≥ 0.60).

All correlations with *p* < 0.05 are considered statistically significant. Bold values denote statistical significance at the *p* < 0.05 level.

## Discussion

This study explored the relationship between BWS and neurodegenerative diseases leading to CI in older adults, suggesting that a decline in BWS is associated with an increased risk of neurodegenerative diseases. A reduction in BWS significantly elevated the risk of neurodegenerative diseases. Furthermore, a BWS threshold of 0.395 m/s effectively differentiated individuals with neurodegenerative diseases from those who were CH. The study also demonstrated that BWS may exhibit distinct neuropsychological impairment profiles across various conditions, including aMCI, AD, PD, and non-AD.

Previous research has highlighted that the risk of rapid cognitive decline was greater in individuals with slow WS than in those with normal WS [33, 34]. The present study extends this line of research by investigating the relationship between BWS and neurodegenerative diseases. Our analysis revealed that a 0.1 unit decrease in BWS increased the prevalence of disease in individuals with aMCI (30%), AD (46%), PD (29%), and non-AD (61%) compared to CH individuals. These findings indicate that a reduction in BWS significantly elevates the risk of neurodegenerative diseases and may serve as a marker of cognitive decline and neurodegenerative processes. The results remained significant even after adjusting analyses, demonstrating the robustness of the findings. Additionally, the highest risk was found in the non-AD group, which may be linked to more disrupted spatiotemporal gait parameters observed in non-AD dementias than in AD dementia [35].

This study determined a cutoff value of 0.395 m/s for BWS in older adults and provided information on analyses regarding sensitivity and specificity values. To the best of our knowledge, this is the first study addressing a threshold value for BWS to differentiate older adults with neurodegenerative diseases causing CI. In the ROC analysis, the AUC value was 0.723, indicating that the BWS is an effective distinguishing feature for detecting these individuals. The high sensitivity (0.72) suggests that the test is unlikely to miss true cases, while the specificity (0.62) indicates that some false positives may occur. The moderate specificity suggests that BWS alone may not be sufficient for definitive discrimination, but may still serve as a clinically useful screening tool. The diagnostic accuracy of BWS was higher in women than in men, which may be explained by neuromuscular and hormonal factors in women [36].

Upon reaching our main results, we identified several significant findings in the meantime that, although not our primary aim, we believe are important to share. We believe these results can provide a foundation for future studies by researchers interested in exploring the relationship between walking speed and cognitive and functional evaluation of older adults. First, in older adults with AD, BWS showed significant associations with both phonemic and semantic VF, while in those with aMCI, BWS was associated only with semantic VF. VF tests, which are commonly used in the assessment of older adults with memory problems, have been established as potential markers for predicting the conversion from aMCI to AD [37, 38]. Given the strong correlation between semantic VF and BWS in the present study, BWS might be a salient predictor of early AD risk in patients with aMCI. Furthermore, the use of BWS to predict the progression from aMCI to AD can offer valuable insights into early cognitive changes, particularly when individuals are illiterate and have low education levels. VF tests can be challenging for grading the cognitive skills of illiterate and low-educated individuals [39]. Thus, we hope that confirmation of this result will provide support for the future utility of BWS as an appropriate tool for creating risk profiles for illiterate and low-educated individuals with memory complaints or those with aMCI at risk of progressing to AD. Second, a significant correlation was found between BWS and BADL in all participants. This result indicates that BWS is not only aligned with cognitive status but also reflects functional status. Thus, a decline in BWS might predict dependency on BADL in older adults. It may be utilized to predict loss of independence in routine clinical practice.

A key strength of the present study lies in its design, wherein gait, balance, cognitive function, and functional status -core components of the geriatric assessment- were evaluated with concurrently administered BWS in older adults. The study focused on the relationship between BWS and the risk of neurodegenerative conditions. The study further highlights the utility of a simple, low-cost test to assess both functional and cognitive status in older adults, eliminating the need for specialized training. A clear diagnostic cutoff of 0.395 m/s was presented, enabling early detection of individuals at risk for neurodegenerative conditions. Another strength is the demonstration of BWS as a candidate practical tool for constructing risk profiles, particularly among illiterate or low-educated individuals with memory complaints or MCI. The findings suggest that BWS has the potential to predict loss of independence.

The study has several limitations. Its cross-sectional design restricts the ability to infer causal relationships between BWS and the diseases, thereby limiting conclusions about whether reduced BWS is a result or a cause of cognitive deterioration. Future longitudinal studies are necessary to clarify this. Additionally, the relatively small sample size of the non-AD group limited the ability to perform subgroup analyses, which may have masked disease-specific gait patterns. Furthermore, the study’s focus on specific diagnostic groups may constrain the generalizability of the findings to the broader older adult population. The analysis did not encompass all cognitive subdomains, raising the possibility that some meaningful associations may have been overlooked. Finally, the internal consistency of the tests was not assessed.

This study demonstrated that BWS may serve as a useful indicator of both functional and cognitive status in older adults. As a simple, rapid, and low-cost clinical test, BWS has a great clinical utility for routine use. Given its potential to reflect cognitive function, gait and balance, and independence in activities of daily living, BWS should be integrated into standard geriatric clinical practice. Prospective studies with larger and more diverse populations are needed to validate and extend the generalizability of these findings.

## Data Availability

The datasets used and/or analyzed during the current study are available from the corresponding author on reasonable request.
